# Humans and cyber-physical systems as teammates? Characteristics and applicability of the human-machine-teaming concept in intelligent manufacturing

**DOI:** 10.3389/frai.2023.1247755

**Published:** 2023-11-03

**Authors:** Franziska Bocklisch, Norbert Huchler

**Affiliations:** ^1^Department of Mechanical Engineering, Chemnitz University of Technology, Chemnitz, Germany; ^2^Fraunhofer Institute for Machine Tools and Forming Technology, Chemnitz, Germany; ^3^Institute for Social Science Research, Munich, Germany

**Keywords:** human-machine-teaming, human-centered artificial intelligence, cognitive engineering, complementarity, shared knowledge and goals, human-centered industry 4.0/5.0

## Abstract

The paper explores and comments on the theoretical concept of human-machine-teaming in intelligent manufacturing. Industrial production is an important area of work applications and should be developed toward a more anthropocentric Industry 4.0/5.0. Teaming is used a design metaphor for human-centered integration of workers and complex cyber-physical-production systems using artificial intelligence. Concrete algorithmic solutions for technical processes should be based on theoretical concepts. A combination of literature scoping review and commentary was used to identify key characteristics for teaming applicable to the work environment addressed. From the body of literature, five criteria were selected and commented on. Two characteristics seemed particularly promising to guide the development of human-centered artificial intelligence and create tangible benefits in the mid-term: complementarity and shared knowledge/goals. These criteria are outlined with two industrial examples: human-robot-collaboration in assembly and intelligent decision support in thermal spraying. The main objective of the paper is to contribute to the discourse on human-centered artificial intelligence by exploring the theoretical concept of human-machine-teaming from a human-oriented perspective. Future research should focus on the empirical implementation and evaluation of teaming characteristics from different transdisciplinary viewpoints.

## 1. Introduction

### 1.1. Paper objectives

The technological evolution toward anthropocentric digitalization at work is rendered possible by new information and communication technologies as well as Artificial Intelligence (AI). It raises the questions: why and where is human-centered AI (HCAI) needed at work? Which recent theoretical concepts and methods can be applied to guide this complex, transdisciplinary endeavor in a responsible way? One good starting point is to clarify what “human-centeredness” means. As this is a very important but also general question, we use it as orientation to identify key characteristics and factors related to the more focused concept of human-machine-teaming (HMT) and apply it to the working field of intelligent manufacturing. HMT can be defined as (1) a form of teamwork between humans and technical systems characterized by “real” interdependency between teammates such as joint activities toward a common goal (Johnson and Bradshaw, 2021). From another – more technical point of view – HMT may be characterized as (2) “the dynamic arrangement of humans and cyber-physical elements into a team structure that capitalizes on the respective strengths of each while circumventing their respective limitations in pursuit of shared goals” (Madni and Madni, 2018; p. 5). As these different transdisciplinary viewpoints on HMT may not be harmonized within one definition, we aim to capture key characteristics and criteria of HMT instead, using a literature review based on scoping method. The identified HMT criteria candidates are discussed and shortly illustrated by two example technologies from the working field of industrial manufacturing (human-robot-collaboration in assembly and intelligent decision support in thermal spraying). Our main objective is to contribute to the discourse on HCAI at work and to advance the development of the transdisciplinary, theoretical concept of HMT. Our comments come from a human-oriented perspective building on the research backgrounds from cognitive and engineering psychology as well as sociology of work and technology.

### 1.2. Human-centered artificial intelligence in industry

**Figure 1:**
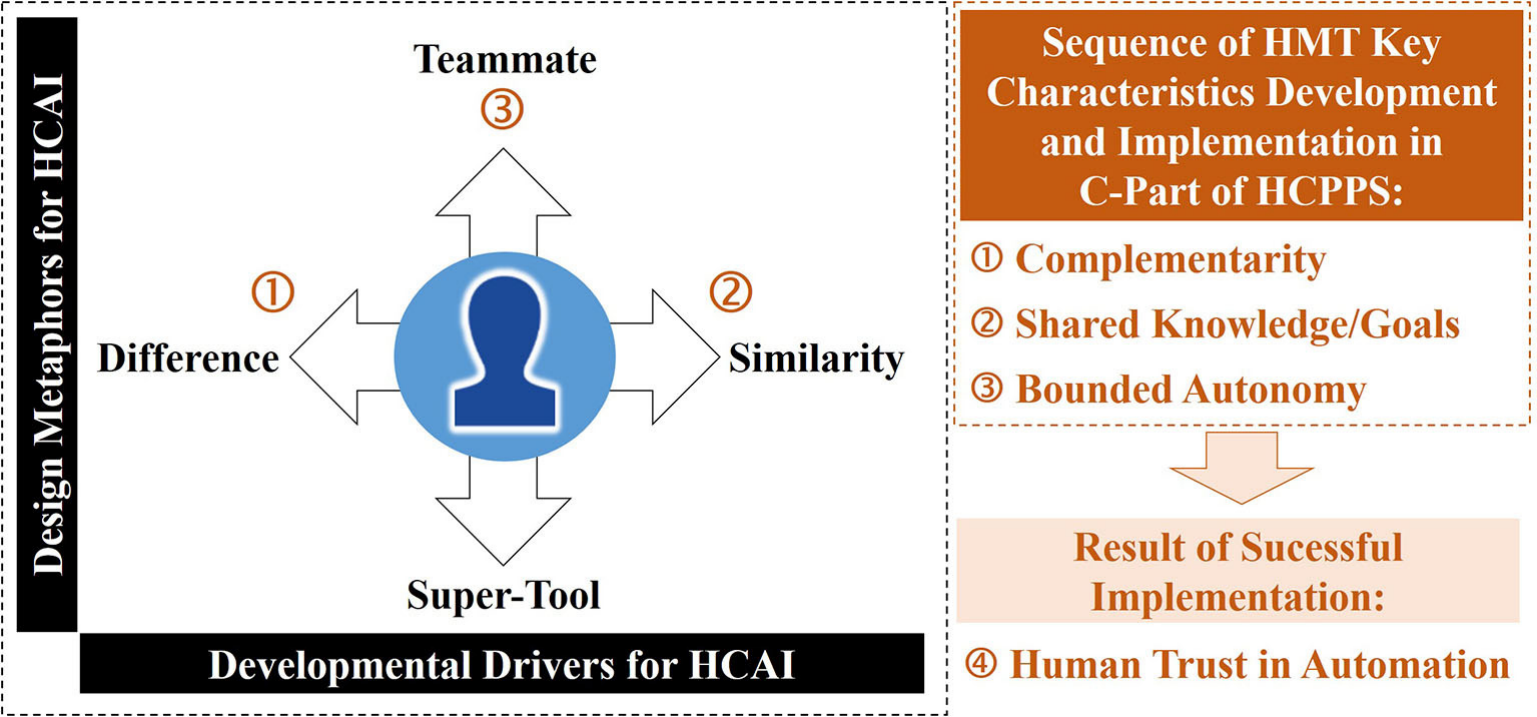
Human-centeredness as resulting balance between different technical design metaphors (**left**; vertical axis) and developmental drivers for Human-Centered Artificial Intelligence (HCAI; horizontal axis). Sequence of Human-Machine-Teaming (HMT) key characteristics development **(right)**. The first three characteristics are especially promising for industrial applications and should be integrated using HCAI in Human-Cyber-Physical-Production-Systems (HCPPS).

Generally, HCAI can be of interest in all areas of work in which complex problems have to be solved and a high level of security, speed, quality or efficiency of human-machine interactions is required. Among the fields are, for instance, military, medicine, mobility, finance, management and administrative knowledge work as well as intelligent manufacturing. The manufacturing industry is one of the most important economic sectors in the industrialized nations with a very high number of employees in various fields of work. The necessity of an anthropocentric perspective within Industry 4.0 is clearly recognized (see Rauch et al., 2020; Eich et al., 2023) and Xu et al. (2021) characterize the next step toward Industry 5.0 with its core values sustainability, resilience and true human-centeredness. Upcoming concepts such as human-cyber-physical systems (HCPS) show, how human-centeredness can be implemented concretely (Lamnabhi-Lagarrigue et al., 2017; Madni and Madni, 2018; Zhou et al., 2019; Bocklisch et al., 2022). HCPS combine three very different system parts: The human (H) in its two roles as user and developer of the technical system. The technical systems consists of (1) the physical subpart (P) controlled by (2) a cyber-system (C). Due to the complexity of manufacturing technologies and production processes, the C-part may implement AI algorithms. They represent effective means for machine control and should be developed toward HCAI (Shneiderman, 2022) and explainable AI (Hagras, 2018; Samek and Müller, 2019) to enable more joint working with humans and suitable support for cognitively demanding working tasks. Keep the human in the loop, is not primarily only a normative demand, but it is argued why this is functional (Huchler, 2022). Thus, humans have a special role in managing complexity in CPS (Böhle and Huchler, 2016). To that end HCPS offers a systemic and transdisciplinary perspective on automation allowing for flexibility and the development of semi-autonomous systems (Madni and Madni, 2018; Bocklisch et al., 2022). As a variety of industrial applications does not comply with the requirements for full automation and, furthermore, agility as well as (social) sustainability became increasingly important facets of modern work, the traditional, linear conceptualization of automation is not expedient. Hence, theoretical concepts for HCAI need to be derived from systemic and maybe even circular socio-technical concepts because (1) the technical developments effect use (and usefulness) of technical systems and the use (or misuse and disuse) has consequences for further developments and (2) automated systems are embedded again in social circumstances such as communication interfaces and work processes (Huchler, 2022). Circular concepts explicitly take into account the emergence of new forms of work or working tasks, being constantly created by automation of processes, systems and system components in various stages of technical development and use. In order to keep the human operator in the loop and combine human strengths with CP-systems capabilities in a complementary way, technical parts and AI algorithms should be developed in close accordance with human objectives and needs. Interests, discourses and narratives of the future drive technological innovations. They are subject to social dynamics between technology promises and disappointments, technological path dependencies, and changing images of man and technology. Recently, “human-centeredness” started to guide AI developments. Depending on the definition of AI used by the developers, the “similarity principle” may address cognitive aspects (e.g., models approximate human thinking or decision-making processes) or behavioral aspects (e.g., the final decision and intelligent machine behavior). Furthermore, the “difference principle” can mean that AI is “more rational than human cognition and behavior” (rational thought/action; cf. Russell, 2010, p. 2). If these different viewpoints in AI definitions are not payed attention to, one may easily misinterpret human-centeredness only as “similar” to the way, humans think, feel or act. However, true human-centeredness arises in the field of tension between the developmental opposites similarity (e.g., constituted by shared knowledge and shared goals; see application example 2.3.2 below) and difference/diversity (e.g., complementarity, non-redundant functions; see 2.3.1). Furthermore, human-centeredness may take different design metaphors as basis for AI and technological developments (cf. Figure 1, inner rectangle). For instance, AI may act as “supertool” or “tele-bot” vs. “intelligent agent” or “teammate” (Shneiderman, 2022). With regard to the chosen work application, we focus here on HMT because this concept may create tangible advantages and foster responsible solutions for industry in the mid-future. Compared to classical automation HMT is a rather transdisciplinary research field, that aims at integrating human-centered aspects into technology development more explicitly. This is done not only on a user-centered design level, but also more deeply, for instance, in the support or automation of cognitive processes (cf. example in Section 2.3.2; Bocklisch et al., 2022). This leads to a shift in goals: the goal of classical automation is to replace the human worker if possible. HMT aims at forming a joint work system with human and cyber-physical parts based on HCAI. It integrates the potentials of both in new productive ways (Huchler, 2022) and may include a high degree of technical automation and human control (cf. Shneiderman, 2022). In the following, we review the concept of HMT with emphasis on finding key characteristics. Thereafter, we discuss the potential of two HMT criteria candidates for two industrial applications: human-robot-collaboration and intelligent decision-support. Other criteria are also reported and commented on. Then, we summarize which ones are (not yet) applicable and ready to be transferred from human-human-teams to human-cyber-physical-teams. Finally, we conclude and summarize future prospects for the HMT discourse and development.

## 2. Human-machine-teaming

HMT aims to transfer characteristics and principles of successful human-human-teams to human-cyber-physical-teams. This raises the question which features (= key characteristics) are ready and worth being implemented by HCAI in HCPS in the working field of production. Based on this, research can be planned into suitable methods and AI algorithms able to implement the identified features in the C-part.

### 2.1. Method

A structured literature review was performed starting with a scoping procedure (e.g., Arksey and O'Malley, 2005) to identify the breadth of contributions in HMT followed by a focused in-depth evaluation of records that present key characteristics of HMT for intelligent manufacturing. We understand key properties to be fundamental features of the theoretical HMT concept that may be addressed or implemented in some way in HCAI technology development in industrial applications in the near or mid-term future. The single keyword was “human-machine-teaming” and research results were limited to English documents between January 1 2016 and 31 May 2023 (no entries before 2016). For identification, the following databases revealed numerous records: scopus (*N* = 102) and Google scholar (*N* = 956). Exclusion and eligibility criteria were deliberately chosen rigorous in the second review phase. It was not the objective of this mini review to exhaustively review the research field of HMT or of related concepts (for this see Damacharla et al., 2018; O'Neill et al., 2022; Greenberg and Marble, 2023). Instead, we aimed to find key characteristics of HMT with sufficient conceptual strengths and high applicability to manufacturing that have already been taken up to a certain extend by the scientific community, to discuss them in-depth in terms of content (see 2.2) and illustrate them with the help of technological examples (see 2.3). After exclusion of redundant records, for 948 documents titles/abstracts were screened to identify eligibility (criterion was HMT definition by key characteristics) for full-text review (remaining *N* = 16 documents). After full text review, the remaining results were selected because they represent groundwork papers (*N* = 3: Brill et al., 2018; Madni and Madni, 2018; Johnson and Bradshaw, 2021). The HMT characteristics mentioned therein are discussed subsequently in the light of HCAI and industrial work context mainly from a cognitive psychology/human factors and work sociological point of view.

### 2.2. Selected key characteristics of human-machine-teaming

According to Madni and Madni (2018), HMT is the dynamic arrangement of humans and CPS into a team structure in pursuit of shared goals. Johnson and Bradshaw (2021) emphasize the interdependence relationship between teammates and point out that a team partner's behavior should be observable, predictable and directable. Brill et al. (2018) summarize the following facets for HMT: (1) complementarity, (2) shared knowledge and shared goals, (3) bounded autonomy, (4) mutual trust and (5) benevolence. Complementarity and shared knowledge/goals are related to how people make sense of situations in the field of tension between difference and similarity (Kelly, 1955). Therefore, these fundamental drivers also influence technical developments (e.g., difference: non-redundant complementary functions of technology compared to human capabilities vs. similarity: representation of human knowledge and goals in technical systems; see Figure 1, left). A meaningful sequence of development of HMT starts with these two criteria. Thereafter, the degree of automation or bounded autonomy of the cyber-part can be increased (see Figure 1, right; third criterion). Human trust in automation results from the transparent and successful implementation of these three characteristics. “Mutual trust” and “benevolence” are not applicable for manufacturing working applications (see Discussion). In the following, we focus on complementarity and shared knowledge/goals (see below) as those facets are already subject of HCAI-oriented research and at least – partly – studied in the context of manufacturing applications. Furthermore, they are prerequisites for bounded/semi-autonomy (Madni and Madni, 2018) and, hence, especially promising to establish a teaming relation.

### 2.3. Relevant aspects of human-machine-teaming in industrial working applications

Two aspects of HMT seem to be of special interest for industrial working applications: complementarity and shared knowledge/shared goals. With the help of two examples – one embodied and one un-embodied, cognitive technology – we outline the potential of these criteria in more detail.

#### 2.3.1. Complementarity in human-robot-interaction

It is quite simple: two people who are able to accomplish the same working task may nevertheless share work and form a team. When a robot can do the same thing as a human team partner this usually results in full automation. Even better, in terms of flexibility and robustness of teamwork, is the combination of partners' abilities that complement each other (Huchler, 2020) and may as well combine non-redundant strengths (Madni and Madni, 2018). Nevertheless, it is favorable if workers and robots have overlaps in their skills in a “mixed skill zone.” This allows for adaptive interaction and may be organized in an AI-based human-centered way (Albu-Schäffer et al., 2023). The more humans and robots complement each other, the more productive interaction works (Huchler, 2022) affecting individual motivation at work in a positive way, for example, toward more effectiveness, empowerment, pride of production (“Produzentenstolz”) and technology appropriation. Consequently, this increases trust in and social attachment to work tools in the second step. Similar to how construction workers feel enabled by an excavator in such a way that they “name” and maybe even “pet” it, collaborative robots can empower their human teammates as well. This feeling of support is based on complementarity and just not on similarity. Building on an extensive research line in industrial sociology on the particular relevance of work action and experiential knowledge in technologized work environments (e.g., Böhle and Milkau, 1988; Pfeiffer, 2007), Huchler et al. (2021) reported results of an extensive study in which the development and deployment process of an innovative robotic system for automated wiring of control cabinets was accompanied over 3 years (Huchler et al., 2021). The technical design approach initially chosen was mimicking the way humans work. It systematically narrowed developmental paths guiding directly toward the objective of full automation. The resulting technical solution was ineffective due to overwhelming complexity and automation limitations. A major problem was that there was no idea for productive worker involvement. As a result, the workers had to wait and repeatedly step in when the robot made mistakes. Furthermore, skill degradation, lack of integration of existing competencies as well as problems with allocation of functions and deployment were observed. The fallback solution after several attempts of correction was the complementary consideration of workers' cognitive and manual competencies resulting in the idea of a “supertool” workplace. The promise of cost savings through robotization was no longer linked to the simple idea of saving labor costs (substituting automation), but to increasing the productivity of existing employees (complementary automation). As a prerequisite for successful support in complex socio-technical contexts and HCPS, the places where people with their specific competencies are needed must be identified. Then socially sustainable and complementary HMT can be established. In this context, it is important to design the interaction as well as the permanent technological transformation in a “co-evolutionary” way so that people and technology can further develop along their different potentials in order to permanently create new complementarity relationships and maintain innovation capabilities (Huchler, 2022). These findings are supported by further qualitative and quantitative research on the relationship between human work capacities and collaborative lightweight robots (e.g., Pfeiffer, 2016, 2018).

#### 2.3.2. Shared knowledge and goals in intelligent decision support for manufacturing

In manufacturing technologies needed for production of daily life goods, humans operate highly complex machines and technical processes such as in forming, welding or coating. Many technologies rely heavily on human expert knowledge and skills and, hence, can and will not be automated completely in the next future. Physical interactions have been improved by safety standards, worker protection and external means such as exoskeletons or use of robots (see above). However, due to technological and AI developments, system complexity increased rapidly shifting loads toward cognitive aspects (Darnstaedt et al., 2022). Hence, operators would benefit from cognitive augmentation and intelligent support for decision-making, problem solving or fault diagnosis. A prerequisite for establishing a connection between a CPS and a human that resembles a human-human team relationship is that the team partners have a common understanding about the shared work task and goals. To achieve this, the knowledge representation in the CPS must be closely aligned with human expert knowledge (cf. Figure 1: similarity principle) to enable transparent understanding and good interactions. Otherwise, there is a risk that the CPS will represent something (e.g., from sensor data) that has no substantive meaning for humans. If this is the case, then there is no good basis for human-centered and joint teamwork, for example, joint decision-making in complex situations. This research gap is recognized and partly addressed with AI for different manufacturing technologies such as coating (Bobzin et al., 2022; Mahendru et al., 2023). These solid domain-oriented research approaches should be enriched by focusing more explicitly on the human perspective. For instance, by considering action-guiding rules for optimization of technical parameters (Venkatachalapathy et al., 2023) or elicitation of domain knowledge and expert mental models (Hoffman, 2008; Andrews et al., 2023). Sharing knowledge and goals in the sense of how a human “shares” ideas with another human is challenging. First, relevant knowledge needs to be elicited. This is possible but only within the boundaries of what can be brought to consciousness (expert-driven approach; Hoffman et al., 2021) or what can be measured and interpreted semantically without doubt (data-driven approach). Nevertheless, it will never be “complete” compared to the human treasure trove of experience, which is continuously growing and can only be described and formalized in parts (Huchler, 2017). Second, the elicited knowledge requires transparent and strictly HCAI to form an interdependence relationship that is mutually explain- and understandable. In order to do so, a combination of different AI algorithms – knowledge- and data-based methods – are needed to ensure compatibility with different human performance levels such as skill-, rule- or knowledge-based behavior (Rasmussen, 1983). Pure sensory- and data-based procedures will not form a sufficient basis for HMT the intelligent manufacturing because they can only grasp a limited area of what is actually necessary (Rasmussen, 1983; Bocklisch and Lampke, 2023; mainly skill-based behavior).

## 3. Discussion

### 3.1. Key characteristics of human-machine-teaming in industrial working applications

HMT is an innovative concept with potential for real-world working domains such as manufacturing. It may guide HCAI developments toward more anthropocentric designs, new forms of work and human-machine interaction. Based on a review of recent literature as well as own preliminary work, we consider the systematization of Brill et al. (2018) as one good starting point for in-depth discussion of potential teaming characteristics for HCAI in industrial manufacturing. In Figure 1, the criteria have been systematized and placed in a meaningful order of development and implementation in HCPPS. Criteria “complementarity” and “shared knowledge/goals” have been illustrated with concrete examples (see above), because (a) they have already been researched to a certain extent in the work context of intelligent manufacturing and (b) they represent essential foundations for criteria “bounded autonomy” and “trust.” In the following, the criteria are discussed in detail, placed in an overall context, and illuminated with regard to future research needs.

(1) Complementarity: yes, in our opinion this criterion is central for HMT because the dissimilarity/diversity facet and may be used to augment humans by powerful complementary functionalities that are provided by the cyber-physical-production-system (CPPS). However, this is not a static concept but characterized as ongoing innovation process – including permanent search for new potentials for complementarity and (re)adjustment of education and further training. Hence, there is need for a better understanding of the differences of human and technology/AI as well as of automation dynamics and changes in the human-technology relationship.

(2) Shared knowledge/goals: These criteria refer to the opposite of complementarity and use similarity principle to constitute a common working basis between humans and CP-systems. A successful and reliable working relation as well as efficient function allocations need shared knowledge and goals. Both, implicit and explicit forms of human knowledge are needed in working contexts. Hence, cognitive engineering methods for knowledge elicitation, structured systematization and transparent AI-implementation need to be developed further. Joint goals can potentially be defined on various levels of abstraction. High-level experts, for instance, persons controlling complex plants, are able to use their rich knowledge hierarchies and related procedures to tackle concrete situations in a very flexible way (Rasmussen, 1983). Changes in the situation are managed by goal or sub goal adaptation. These human strategies to control real-world complexity and act under uncertainty need to be mirrored – at least partly – in the cyber-teammate as well. If this can be achieved successfully will depend on the development of AI regarding adaptivity and learning (e.g., evolving intelligent systems: Angelov et al., 2010; Bocklisch et al., 2017) as well as cognitive transparency and understandability of AI algorithms (e.g., Weller, 2019).

(3) Bounded autonomy: autonomy is always limited and negotiated in social contexts. For HMT, different kinds of autonomies have to be integrated similar to the different “intelligences” (human vs. artificial). The simple technical levels of autonomy (e.g., functionality within a limited context) do not correspond to the complexity of the socially negotiated understanding of autonomy of individuals. As with intelligence, the complexity of the social counterpart is completely underestimated or taken too simplistically. Hence, profound conceptual research should relate theoretical concepts to concrete application examples. This is also necessary because autonomy is a “provocative” criterion that may easily lead to conflicting viewpoints (Brill et al., 2018) as well as fears from the human user side. Technology assessments that evaluate dangers (see “The janus face of autonomy” in Brill et al., 2018) as well as possibilities and derive regulatory principles (Shneiderman, 2020) are therefore needed as well.

(4) Mutual trust and (5) benevolence: Trust is central to establish a successful and harmonic relationship in human-human work teams. One classic definition originates from Lee and See (2004; p. 54): trust is “… the attitude that an agent will help achieve an individual's goals in a situation characterized by uncertainty and vulnerability.” In this respect, it is a good candidate criterion worth being thought of concerning its transferability to HM-teams and closely related to “shared goals” – a part of the definition and thus a necessary condition for trust. Trust in automation is extensively studied (e.g., Lee and Moray, 1992; Hoff and Bashir, 2015; Schaefer et al., 2016; Kohn et al., 2021) and a highly important factor for user-centered design to avoid misuse, disuse or abuse of technology (Parasuraman and Riley, 1997; Lee and See, 2004). Nevertheless, “trust is a complex and nebulous concept” (Hoffman et al., 2013, p. 84) and should not be understood in a too simplistic way as a “lack of information” but rather as a complex process of (reciprocally effective!) establishing the ability to act even beyond (risk) calculations (Huchler and Sauer, 2015). Furthermore, it seems only applicable from a human point of view: a human trusts a robotic system or a suggestion of a decision support system (more precisely: the people and institutions behind). The relation cannot simply be reversed and named “trust” because trust presupposes physical and/or mental vulnerability, which applies to technology only to a very limited extent. Sociological aspects are important to consider as well. What is often perceived as “trustful relationship” to a technical artifact (similar to a person) is in reality based on social processes (Mayer et al., 1995) in a complex social-technical setting primarily also related to trust in the institutions responsible for technology. This explains some experimental results concerning “over trust” in robots (Aroyo et al., 2021). The institutions and regularities are important guarantors for safety. At least in work contexts, it is evident that trust in and acceptance of technology can be generated much more clearly through utility and empowerment than through similarity which is only one of the polar development drivers (cf. Figure 1). From the human user perspective, too close similarity to human skills comes with a latent threat: substitutability – the opposite of benevolence, which is in our opinion no primary target criterion for HMT. “Mutual” trust and benevolence are no purposeful facets for HMT because technology is not able to trust or act benevolent. Here, the distinction between system trust and personal trust is crucial (Luhmann, 1979). Nevertheless, suitable objective criteria from the technical point of view have to be developed instead.

### 3.2. Limitations and future prospects

Our main objective was to contribute to the discourse on HCAI by having a closer look on the theoretical concept of HMT in the context of industrial work applications. This is intended to be an impulse from a human-oriented perspective on AI developments for future transdisciplinary discourses. Of course, there are many other perspectives on this topic that are equally interesting, relevant and necessary. For example, concepts and empirical work from research on human teamwork (e.g., concerning suitable definitions of “team” and types of teams) and team performance as well as from (software) engineering are crucial for complementing and validating HMT criteria. Here, our focus was on theoretical considerations but guides on the implementation of HMT aspects already exist, highlighting the practical relevance of the topic (e.g., McDermott et al., 2018). Industry 4.0/5.0 developers would benefit from operationalizing various HMT criteria in industrial examples. Not only on the general level of user-centered design guides but more in-depth for specific technical applications (Bocklisch et al., 2022). Another limitation was the narrow scope of search terms: given the huge number of literature and our specific goal to find applicable key characteristics for manufacturing and comment them in the light of two short application examples, we only selected “HMT” as keyword for scoping review. Other words, such as “human-autonomy-teaming,” “human-agent-teaming,” “human-machine-interaction,” “human-machine-symbiosis,” and many thematically related terms in various combinations would lead to a more comprehensive and – concerning the vast body of empirical evidence – less biased summary (cf. O'Neill et al., 2022). Furthermore, we did not discuss all potential HMT-criteria as key features but reduced to five aspects from which we selected two to outline their concrete potential for industrial applications with the help of two technical examples. On the one hand, this specific procedure and scope resulted from the fact that some facets clearly need to be given ex ante to be of interest for HCAI (such as observability; cf. 2.3.2 and boundaries of human knowledge elicitation and data acquisition from human sources). On the other hand, this was because some criteria are very similar and somehow eclectic (e.g., bounded autonomy vs. semi-autonomy or interdependency). Whether these slightly different connotations of criteria, e.g., of the core characteristic ”bounded autonomy,“ should be taken into account cannot be adequately assessed at present. This will be shown by the operationalization of the characteristics in the empirical work, the practical application and the evaluation of these results.

In conclusion, HCAI has a large potential to promote new types of human-machine-interaction at work, such as outlined here in parts for HMT. The transfer of some characteristics of HH-teams to HCP-teams are promising and feasible for real-world working contexts such as intelligent manufacturing, others not – because humans and technology are very different in nature (Madni and Madni, 2018; p. 4f) – or not yet – because HCAI capabilities still need to be developed further. If HMT capabilities are to be integrated into technology development of HCPS as a concrete form of HCAI, then the start could – in our opinion – be to establish complementarity and shared knowledge/goals. Thereafter, the effects of this development should be evaluated from different viewpoints that are important in intelligent manufacturing such as human-oriented criteria (e.g., user acceptance, mental workload), technical or business oriented aspects (e.g., system performance, product quality, resource efficiency and costs).

## Author contributions

All authors listed have made a substantial, direct, and intellectual contribution to the work and approved it for publication.
